# Orientation and Microstructure Evolution of Al-Al_2_Cu Regular Eutectic Lamellar Bifurcating in an Abruptly Changing Velocity under Directional Solidification

**DOI:** 10.3390/ma13041004

**Published:** 2020-02-24

**Authors:** Ka Gao, Zan Zhang, Junliang Zhao, Dejian Sun, Fu Wang

**Affiliations:** 1 School of Materials Science and Engineering, Zhengzhou University of Aeronautics, Zhengzhou 450015, China; 2 Henan Key Laboratory of Aeronautical Material and Application Technology, Zhengzhou 450015, China; 3 School of Mechanical Engineering, Xi’an Jiaotong University, Xi’an 710049, China

**Keywords:** directional solidification, solidification rate, lamellar bifurcation, grain growth, growth orientation

## Abstract

In an abruptly changing velocity under directional solidification, microstructures and the growth orientation of Al-Al_2_Cu eutectic lamellar were characterized. The change in solidification rate led to an interfacial instability, which results in a bifurcation of the eutectic lamella into new, refined lamellae. The growth orientation of the eutectic Al_2_Cu phase was also only in its (001) direction and more strongly oriented to the heat flow direction. The results suggest that the eutectic lamellar Al-Al_2_Cu bifurcation and the spacing adjustment may be caused by the rate determining lateral diffusion of the solutes after interfacial instability.

## 1. Introduction

According to current research, the solidification process of eutectic alloy [1,2,3,4,5,6], including regular and irregular eutectic, is complicated because the solidification microstructures and phase characteristics are closely related to the coupling growth of eutectic phase. For regular eutectic alloy growth, more attention has been paid to the growth evolution of regular eutectic microstructure under directional solidification, such as the selection mechanism of regular eutectic lamellar spacing, the corresponding relationship among solidification rate, undercooling and morphological transformation [7,8]. Among them, the lamellar space changing is the main and direct influence factor, which affects the change of the microstructure and the properties of the eutectic alloy [8]. While, in the process of unsteady-state solidification, a regular eutectic microstructure was easy to be affected by kinetics, and corresponding interface instability phenomena would be occurred. Then the lamellar spacing was changed by bifurcating or merging in the eutectic phase of at an abruptly changing velocity. Many scholars have done a lot of research on it. For example, on the basis of the Jackson–Hunt model [9], Cline [10,11,12] considered that the lamellar spacing changes were related to the local interface changing due to eutectic solidification interface instability. While, Strassler [13] et al. held that the maximum laminar spacing was determined by temperature gradient. Then Mollard [14,15] and Trivedi [16] believed that the adjustment of lamellar spacing was mainly accomplished by the defects movement. But Double et al. [17] thought that the adjustment of lamellar spacing of directionally solidified Al-Al_2_Cu eutectic was realized by homogeneous nucleation of lamellar phase because of the abruptly changing interface instability. So until recently, there was no uniform understanding of the mechanism for forking and merging the regular eutectic lamellar.

Due to the final morphology determined by orientation changing during crystal growth, the spacing adjustment was closely related to the growth orientation of eutectic phase. Therefore, many scholars had also studied the crystallography of Al-Al_2_Cu eutectic [18,19,20,21] and obtained some different results about eutectic lamellar growth direction and inertial interface, in order to clarify the characteristics of lamellar coupling growth. However, until now there have been no finding on the growth orientation near the abruptly changing interface instability, which determines the growth and final microstructure of eutectic crystal. Moreover, whether it is bifurcation or re-nucleation of regular eutectic lamellar spacing adjustment, the growth direction before, and after, the spacing change is still unclear, which is unfavorable for further understanding the coupled growth process of eutectic lamellar.

Thus, in this work, the three-dimensional (3D) microstructures evolution and growth orientation of Al-Al_2_Cu eutectic lamellar in an abruptly changing rate during directional solidification were investigated. Moreover, the deviation relationship between the growth orientation of eutectic Al_2_Cu phase and the heat flow direction was characterized and analyzed by the micro orientation analysis methods, in order to determine the variation of growth direction before and after the lamellar spacing was adjusted and changed. Then, the brief description about spacing adjustment mechanism of eutectic lamellar forking was given. Through this study, new research ideas and experimental references can be provided for the regular eutectic growth process.

## 2. Experimental Procedures

### 2.1. Materials

Al-Al_2_Cu eutectic alloy with 33.2 wt.% Cu was prepared in a vacuum induction melting furnace with purity aluminum (99.95 wt.%) and copper (99.9 wt.%). All chemical reagents and raw materials were purchased from Kaitong Chemical Reagent Co. Ltd., Tianjin, China and Aluminum Corporation of China Co. Ltd., Beijing, China. The alloy sample were enveloped in the high purity Al_2_O_3_ tube with an inner diameter of 7 mm and a length of approximately 150 mm. Then, the samples were heated by a graphite heater at 700 °C, and then held isothermal for 30 m using a Bridgman vertical vacuum furnace (DS-1600, Haozhiduo New Materials Technology Co., Shenyang, China), in order to homogenize the original ingot composition. Subsequently, the samples were firstly moved downwards at 2 μm/s reached directional solidification distance 50 mm, then pulled at abruptly changing velocity at 100 μm/s for 50 mm. When the directional solidification distance reached 50 mm, the sample was quenched into a liquid Ga-In-Sn pool to keep the S/L interface.

### 2.2. Characterization 

To obtain the SEM sample, the directionally solidified samples were then cut along a transverse-section. The microstructures of the polished specimens were revealed with the agent of H_2_O (46 mL) + HNO_3_ (3 mL) + HF (1 mL) for about 15 s. And then scanning electron microscopy (SEM, JSM-7001F, JEOL Ltd., Tokyo, Japan) was employed to photograph the specimen microstructures. The growth orientations of Al_2_Cu phase in eutectic were measured by the electron back-scattered diffraction (EBSD) in scanning electron microscopy (SEM, Zeiss Supra 55, Carl Zeiss AG, Jena, Germany) equipped with the Channel 5 EBSD system (HKL Technology-Oxford instrument, Oxford Instrument Co, Oxford, UK). The EBSD samples were electro-polished at room temperature, in which 5% perchlorate alcohol was chosen as an electrolyte, the voltage parameter was 30–40 V and the time was 15–30 s. Then EBSD scanning step was 0.2 μm. Moreover, by using the serial sectioning technique, the Materialise’s interactive medical image control system (Mimics) software was applied to reconstruct the three-dimensional (3D) microstructures images of the eutectic phase in this work.

## 3. Results and Discussions 

**Figure 1:**
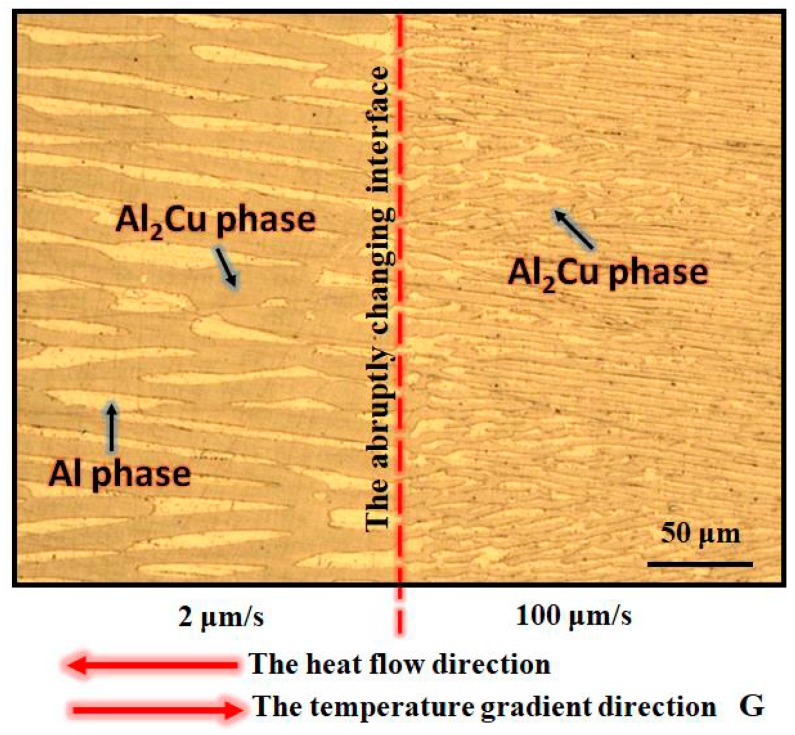
Longitudinal microstructures of the Al-Al_2_Cu eutectic alloy at the abrupt change in pulling rate from 2 μm/s to 100 μm/s.

Figure 1 was the longitudinal microstructure (2D) of the Al-Al_2_Cu alloy when the pulling rate varied from 2 μm/s to 100 μm/s. The abruptly changing interface could be obviously observed. It is well known that the solidification microstructures of Al-33.2%Cu alloy consist of eutectic (Al/Al_2_Cu) based on the Al-Cu phase diagram. So the microstructure was regular eutectic lamellar at a pulling rate of 2 μm/s, which consisted of Al_2_Cu phase [22]. The size of eutectic lamellar was a little larger result in the larger lamellar spacing shown in left of Figure 1. Moreover, the growth direction of eutectic was most along, but not completely parallel to the heat flow direction and the temperature gradient direction. While, there was also regular eutectic (Al/Al_2_Cu) lamellar when the pulling rate was abruptly changed to 100 μm/s. Different with the left eutectic microstructure, after the pulling rate abruptly changed, the size of lamellar and lamellar spacing were obviously decreased rapidly. In addition, through the abruptly changing rate the growth direction of eutectic was also not completely parallel to the heat flow direction and the temperature gradient direction. However, the deviation between them seemed decreasing. 

**Figure 2:**
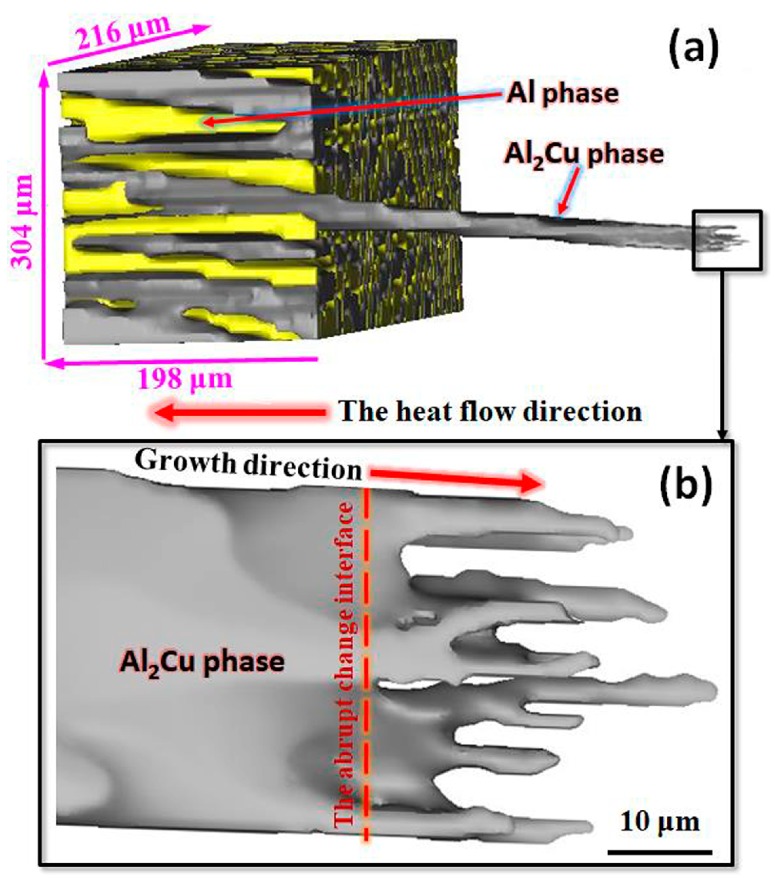
(**a**) The three-dimensional (3D) microstructure of the directionally solidified Al-Al2Cu eutectic in the abruptly changing, and (**b**) the corresponding local enlargement.

In order to further investigate the eutectic spacing adjustment process, the three-dimensional (3D) microstructure of eutectic phase on the abruptly changing interface were reconstructed by the serial sectioning technique [23,24], as shown in Figure 2. The yellow part was Al phase lamellar and the dark gray part was Al_2_Cu phase lamellar in Figure 2a, which growth coupling together. The growth direction of eutectic was not completely parallel to the heat flow direction. After the pulling rate suddenly increased to 100 μm/s, there might be interface instability. Then eutectic Al_2_Cu phase branch began to fork repeatedly in Figure 2b. While, the continuous forking process was not completed at the same place and the abrupt changing interface in Figure 2b. The phase splitting, the inner concave and the branching process of the eutectic phase were observed at a different place. It could be continuously carried out in a completely three-dimensional behavior. The same forking process was true for the eutectic Al phase. Therefore, the Al/Al_2_Cu eutectic spacing adjustment was ongoing in three-dimensional space, which was different from the simple two-dimensional eutectic spacing adjustment. The above results indicated that the three-dimensional (3D) microstructure of eutectic phase in this work could be more clearly shown the eutectic lamellar growth process [20,22]. Through Al and Al_2_Cu phase continuous forking, the size of eutectic lamellar was obviously decreased and Al/Al_2_Cu eutectic spacing was also reduced rapidly. In addition, after the pulling rate abruptly changed, the growth direction of eutectic was also not completely parallel to the heat flow direction. On the basis of the above results, we know that the instability of the eutectic interface caused the two phases to adjust the lamellae bifurcation in three-dimensional space. Its size and spacing were reduced, and then the regular lamellar microstructure was refined quickly. While, the growth behavior may be also responsible for those regular lamellar microstructures.

**Figure 3:**
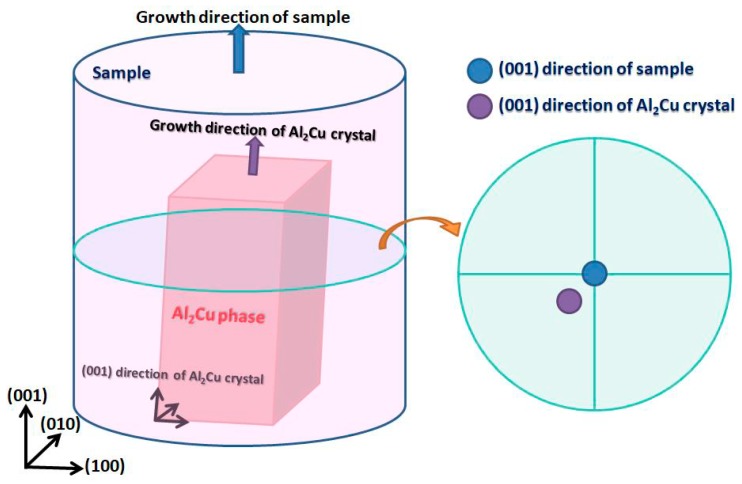
Schematic diagram of (100)-pole figure analysis of sample orientation.

In order to further study the growth process of eutectic lamellae, the growth orientations of Al_2_Cu phase in eutectic in an abruptly changing under directional solidification were investigated and characterized by the EBSD analysis [25]. First, the schematic diagram of (100)-pole figure analysis of sample orientation by EBSD test was shown in Figure 3. When the crystal was grown inside the sample, its growth direction was different from that of the sample. That is to say, there were two coordinate systems in space for crystal and sample. The (100)-pole figure was taken the sample coordinate system (100) as the polar axis, and then investigated the orientation relationship of the crystal coordinate system in the sample coordinate system. Therefore, the growth direction of Al_2_Cu phase was its (001) direction [22], and the growth direction of the sample was (001) axial direction also the heat flow direction. It can be simply considered that when the cross-section of the left figure was the (100)-pole figure in Figure 3. The central position of the pole figure was the (001) axial direction of the sample, and the purple position in the figure was the (001) growth direction of Al_2_Cu phase. The deviation angle between the (001) growth direction of Al_2_Cu phase and (001) axial direction of the sample could be reflected by their distance.

**Figure 4:**
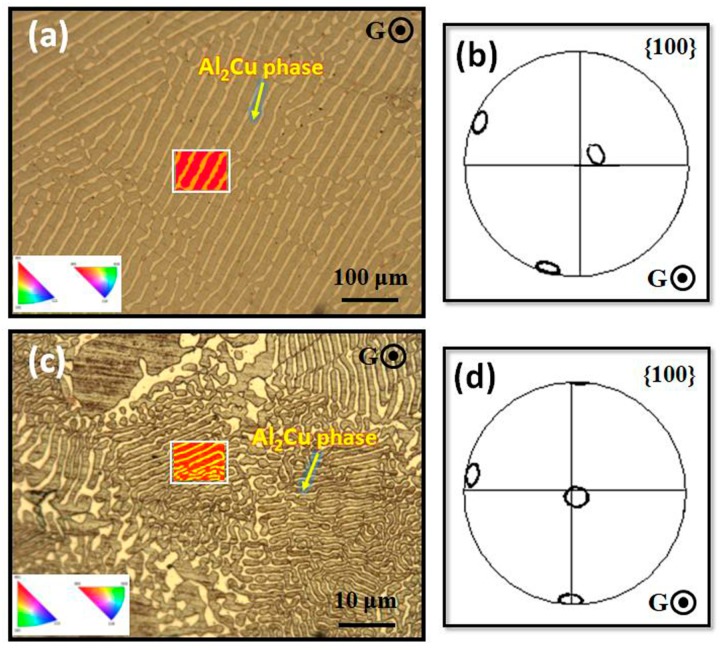
The EBSD analysis of the Al-Al_2_Cu eutectic alloy at the abrupt change in pulling rate from 2 μm/s to 100 μm/s. (**a**) the transverse-section microstructure and (**b**) the corresponding (100)-pole figure of eutectic Al_2_Cu phase at 2 μm/s, respectively; (**c**) the transverse-section microstructure and (**d**) the corresponding (100)-pole figure of eutectic Al_2_Cu phase at 100 μm/s, respectively.

On that basis, Figure 4 shows the EBSD maps in the transverse section, the corresponding (100)-pole figures of Al_2_Cu phase at the abrupt change in pulling rate from 2 μm/s to 100 μm/s, respectively. The regular eutectic lamellar microstructure could be observed at solidification rate of 2 μm/s in Figure 4a. From the pole figure, it was easily deduced that Al_2_Cu phase in eutectic had oriented with its (001) crystal direction. The (001) direction was almost near the heat flow direction in Figure 4b. Then, the deviation angle between (001) direction of Al_2_Cu phase and the heat flow direction were about 10.57°. After the abrupt velocity, the microstructure was also the regular eutectic lamellar. The main growth orientation of eutectic Al_2_Cu phase was also its (001) direction at 100 μm/s in Figure 4c; as observed in Figure 4d, which has been historically relevant. The deviation angle between its (001) direction and heat flow direction were about 8.02°, and the growth orientation of Al_2_Cu phase was further closed in the heat flow direction after the abruptly changing. That agreed well with the 2D and 3D microstructure results in Figure 1 and Figure 2. The above results were indicated that the growth orientation of eutectic was not changed whether or not it bifurcated directly or re-nucleated near the abruptly changing interface instability. There was no other lateral growth direction existing. The growth direction may promote the lamellar spacing adjustment after the interface instability at the abrupt change velocity.

**Figure 5:**
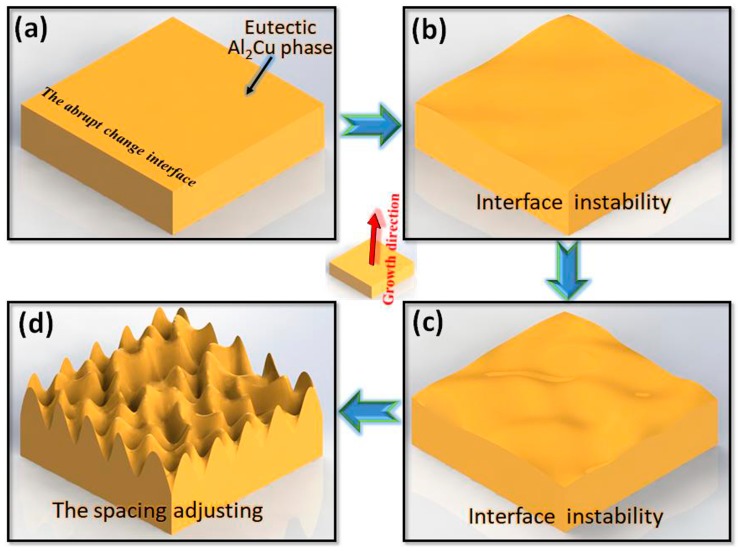
Schematic diagram of the Al-Al_2_Cu eutectic lamellar bifurcating and spacing adjustment: (**a**) Normal solidification rate, (**b**) lower solidification rate, (**c**) near the abruptly changing interface, and (**d**) higher solidification rate.

In this work, we gave a brief illustration on Al-Al_2_Cu regular eutectic lamellar bifurcating when the velocity was changed abruptly under directional solidification. First, before abruptly changing, the solid-liquid interface was stable, as shown in Figure 5a. At this stage, the size of eutectic Al_2_Cu phase was larger, resulting in larger spacing between eutectic lamellar. The eutectic Al_2_Cu phase grew along its (001) direction neat the heat flow direction. With the velocity changing, eutectic interface was easily affected by kinetics and began to be more unstable, as in Figure 5b [26,27]. Then, the solid-liquid interface instability became increasingly obvious (Figure 5c), and fluctuate and bifurcate to form a new interface, leading to solute enrichment and the interfacial surface energy difference. Finally, at the position far from the abruptly changing interface, the new interface would be moved forward and formed the new lamellar in Figure 5d. While, the rate of atoms deposition on different crystal planes changed based on the results on solute enrichment, When the Al_2_Cu phase was grown, solute atoms were aggregated easily on the crystal plane with higher interfacial surface energy, which resulted in the anisotropy of the interfacial surface energy. The anisotropy of interfacial surface energy gradually determined the Al_2_Cu phase growth. However, the transverse interface energy of the Al_2_Cu phase was smaller, which resulted in the hindrance of the lateral solute diffusion. Then, that was not conducive to atom deposition and the new lateral interface growth. While, the (001) growth orientation of the Al_2_Cu phase began to deflect to the preferred orientation. Therefore, the new lamellar continued to grow along its (001) direction, and was closer to the heat flow direction, resulting in smaller lamellar spacing. During the whole process, no other lateral growth direction of eutectic lamellar appeared. This work indicated that when the eutectic lamellar was refined because of the hindrance of the lateral solute diffusion after the interface instability, and then the solid-liquid interface of eutectic mainly moved along the heat flow direction [28]. 

## 4. Conclusions

The microstructure and growth orientation of the Al-Al_2_Cu eutectic lamellar spacing were investigated in an abruptly changing velocity during directional solidification. The deviation relationship between the growth orientation of eutectic Al_2_Cu phase and the heat flow direction was characterized and analyzed by the micro-orientation (the electron back-scattered diffraction) analysis methods. The study conclusion is as follows:

(1) After the abruptly changing interface, Al-Al_2_Cu eutectic lamellar spacing decreased. Al_2_Cu phase was bifurcated to form the new lamellar to refine in the three-dimensional microstructures.
(2) The growth orientation of eutectic Al_2_Cu phase also grew along its (001) direction near the heat flow. The deviation angle between growth orientation of eutectic Al_2_Cu phase and the heat flow direction was decreased after the abruptly changing interface by micro-orientation analysis methods.
(3) A brief description about the spacing adjustment mechanism of eutectic lamellar forking was given. The eutectic lamellar refined was due to the hindrance of the lateral solute diffusion after the interface instability, and the solid-liquid interface of eutectic mainly moved along the heat flow direction.

## Data Availability

The raw/processed data required to reproduce these findings cannot be shared at this time as the data also forms part of an ongoing study.
